# Prevalence, Antimicrobial Resistance, and Risk Factors of Enteric Bacterial Infections Among Children Under Five in Nairobi County, Kenya

**DOI:** 10.1155/ijm/6093453

**Published:** 2025-11-24

**Authors:** Davis Kimile, Perpetual Ndung'u, Peter Karanja, Gervason Moriasi

**Affiliations:** ^1^Department of Medical Laboratory Sciences, School of Biomedical Sciences, Jomo Kenyatta University of Agriculture and Technology, Nairobi, Kenya; ^2^Department of Biochemistry, Microbiology, and Biotechnology, School of Pure and Applied Sciences, Kenyatta University, Nairobi, Kenya; ^3^Department of Medical Biochemistry, Medical School, Mount Kenya University, Thika, Kenya

**Keywords:** antimicrobial resistance (AMR), children under five, enteric bacterial infections, multidrug resistance (MDR), water, sanitation, and hygiene (WASH)

## Abstract

**Background:**

Diarrheal disease remains a major cause of morbidity and mortality in children under 5 years, with the highest burden in sub-Saharan Africa. This study investigated the bacterial etiology, antimicrobial resistance (AMR), and risk factors in childhood diarrhea in Nairobi, Kenya.

**Methods:**

A cross-sectional study was conducted among 216 children aged 0–60 months presenting with diarrhea at hospitals in Nairobi County. Stool samples were cultured and isolates tested against eight antibiotics. Caregiver questionnaires assessed risk factors. Associations were evaluated using Fisher's exact test and odds ratios (ORs) were calculated to determine effect sizes.

**Results:**

A total of 204 bacterial isolates were recovered. *Escherichia coli* was the most frequent (38.2%), followed by *Shigella sonnei* (11.8%) and *Enterobacter cloacae* (9.8%). The overall prevalence of enteric bacterial infection was 94.4%. Key risk factors significantly associated with infection included the absence of consistent soap availability (OR: 7.4, 95% CI: 3.7–15.0), visible poor sanitation (OR: 4.8, 95% CI: 1.7–13.8), and lack of safe drinking water (OR: 12.9, 95% CI: 1.7–98.9). AMR was widespread, with 82.8% of isolates resistant to at least one antibiotic and 58.3% classified as multidrug-resistant (MDR). High resistance to ampicillin and amoxicillin–clavulanic acid was common, exceeding 70% in key pathogens like *E. coli* and *E. cloacae*. In contrast, meropenem, amikacin, gentamicin, and ciprofloxacin retained near-complete efficacy.

**Conclusion:**

Childhood diarrhea in Nairobi is predominantly bacterial, driven by diverse pathogens and exacerbated by poor hygiene and alarmingly high AMR. Urgent integrated interventions are needed, including strengthening WASH infrastructure, promoting rational antibiotic use, and enhancing AMR surveillance. These findings provide a critical baseline for public health action and underscore the need to safeguard last-line antibiotics.

## 1. Introduction

Diarrheal diseases remain a major global public health concern, disproportionately affecting children under 5 years of age due to their immature immune systems and heightened vulnerability to infection [1]. Globally, diarrhea ranks as the third leading cause of mortality in this age group, accounting for an estimated 443,832 deaths annually, with the greatest burden observed in South Asia and sub-Saharan Africa [2]. In Kenya, diarrhea is the third leading cause of pediatric mortality after malaria and pneumonia, contributing to approximately 100 deaths daily [3]. The burden is particularly pronounced in urban informal settlements, such as those in Nairobi County, where overcrowding, inadequate access to safe water, and poor sanitation amplify the risk of transmission [4].

Enteric bacterial pathogens, including *Escherichia coli*, *Shigella* spp., and *Salmonella* spp., are key etiological agents of diarrheal disease. However, their local prevalence, antimicrobial resistance (AMR) patterns, and distribution across Nairobi's heterogeneous urban environments remain poorly characterized [3]. The growing threat of AMR, particularly multidrug-resistant (MDR) strains, further complicates clinical management by diminishing the efficacy of first-line antibiotics [5]. Reports of MDR enteric pathogens circulating in informal settlements such as Mukuru [6] underscore the urgent need for enhanced, context-specific AMR surveillance to guide antimicrobial stewardship and infection control.

Although previous studies in Kenya have documented the prevalence of enteric pathogens and AMR in specific facilities or regions, no comprehensive, population-based AMR profiling has been conducted in Nairobi's pediatric population. The complex interplay between microbial, sociodemographic, and environmental factors within the city's informal settlements remains insufficiently explored [4, 7]. Moreover, the absence of contemporary, locally generated AMR data constrains evidence-based public health interventions and clinical decision-making [5, 8].

This study therefore provides the first comprehensive AMR profile of enteric bacterial pathogens among children under 5 years of age in Nairobi County, Kenya. Specifically, it investigates the prevalence and antimicrobial susceptibility of key enteric bacteria while examining sociodemographic, environmental, and behavioral determinants of infection. By generating a robust, locally relevant dataset, this study is aimed at informing targeted clinical management strategies and guiding public health interventions designed to reduce the burden of diarrheal disease in high-risk urban settings.

## 2. Materials and Methods

### 2.1. Study Area

This study was conducted in Nairobi County, Kenya, which comprises urban, periurban, and informal settlement areas characterized by significant socioeconomic and environmental heterogeneity. Six high-volume health facilities were purposively selected to represent both formal and informal settlements: Gertrude's Children's Hospital (comprehensive referral pediatric center), Mbagathi District Hospital (tertiary government hospital), Kahawa West Health Centre (tertiary hospital), Westlands Health Centre (tertiary private hospital), Mathare North Health Centre (a primary care Level 3 hospital), and Korogocho Health Centre (a Level 3 basic health center). These facilities were chosen due to their high pediatric diarrheal caseloads. All laboratory analyses were conducted at the Microbiology Laboratory of Kenyatta University Teaching, Research, and Referral Hospital (KUTRH), which is equipped for advanced bacteriological and AMR testing.

### 2.2. Study Design and Population

A hospital-based cross-sectional study was conducted between 15th April 2025 and 18th July 2025 to determine the prevalence, AMR patterns, and associated factors of enteric bacterial infections among children aged 0–60 months presenting with acute diarrhea. Diarrhea was defined as the passage of ≥3 loose or watery stools within 24 h. Accompanying parents or legal guardians were also recruited to provide sociodemographic, environmental, and behavioral information through structured interviews.

### 2.3. Inclusion and Exclusion Criteria

Children were included in this study if they were between 0 and 60 months of age, had diarrhea (defined as passing three or more loose or watery stools within 24 h), lived in Nairobi County, and their caregivers provided written consent and agreed to stool sample collection. Children were excluded if they had taken antibiotics within the previous 7 days, if they had serious health conditions such as chronic digestive disorders or severe malnutrition, or if a stool sample could not be collected.

### 2.4. Sample Size Determination

The sample size was calculated using Cochran's formula for cross-sectional studies [9]:

 $$\begin{array}{c} N=\frac{Z^{2}P\left(1-P\right)}{d^{2}} \end{array}$$

where *N* is the required sample size, *Z* = 1.96 (95% confidence interval [CI]), *P* = 0.15 (estimated diarrhea prevalence in children aged ≤5 years in Nairobi County) [3, 10], and *d* = 0.05 (precision).

The calculated sample size was 196, increased by 10% to 216 to account for potential nonresponses. Allocation across facilities was proportional to their average monthly diarrheal caseloads.

### 2.5. Sampling Technique

A multistage sampling strategy was employed [11]. First, the health facilities were stratified by location (urban, periurban, and informal). Within each facility, systematic random sampling was used in outpatient departments. Based on daily attendance, every third (*k* = 3) eligible child with diarrhea was recruited until each facility met its quota (Table 1).

### 2.6. Data Collection

#### 2.6.1. Clinical and Sociodemographic Data

Clinical data, including symptoms, dehydration status, fever, and vomiting, were obtained through clinician assessment and caregiver interviews. Vaccination status of the children was confirmed via child health cards where available. Caregivers completed structured questionnaires capturing sociodemographic (age, sex, education, and income), environmental (water source, sanitation), and behavioral (handwashing, food hygiene, and waste disposal) characteristics.

#### 2.6.2. Stool Collection and Laboratory Analysis

Fresh stool samples (5–10 g) were collected in sterile, wide-mouthed, leak-proof containers labeled with unique identifiers. For children unable to provide stool, rectal swabs were obtained using sterile, premoistened cotton swabs. All the samples were placed in Cary-Blair transport medium, maintained at 2°C–8°C, and transported to KUTRH within 2 h.

### 2.7. Laboratory Analysis

#### 2.7.1. Culture and Bacterial Identification

The stool samples were homogenized in sterile phosphate-buffered saline (PBS). Direct inoculation was performed onto MacConkey agar (Oxoid, United Kingdom) and *Salmonella–Shigella* (*SS*) agar (Oxoid), while enrichment was conducted using Selenite F broth (Oxoid) incubated aerobically at 37°C for 6–8 h, followed by subculture onto *SS* agar. The plates were incubated at 37°C for 18–24 h. Characteristic colonies were subjected to Gram staining using standard protocol (crystal violet 1 min, iodine 1 min, alcohol decolorization 5 s, safranin counterstain 30 s) and a series of biochemical tests: triple sugar iron (TSI) agar for carbohydrate fermentation patterns, IMViC tests (indole, methyl red, Voges-Proskauer, citrate) for *E. coli* confirmation, urease test to exclude *Proteus* species, and oxidase test to differentiate *Pseudomonas* spp. The isolates were further identified using the VITEK2 AST-GN83 card on the VITEK2 automated system (bioMérieux).

#### 2.7.2. Antimicrobial Susceptibility Testing (AST)

In this study, AST was performed using the Kirby–Bauer disc diffusion method on Mueller–Hinton agar (Oxoid) according to the Clinical Laboratory Standards Institute (CLSI) 2022 guidelines [12]. The following antibiotics (*μ*g/disc) were tested: ampicillin (10), amoxicillin–clavulanic acid (20/10), ceftriaxone (30), ciprofloxacin (5), gentamicin (10), nitrofurantoin (300), and meropenem (10). Zone diameters were interpreted as susceptible, intermediate, or resistant per CLSI breakpoints. The minimum inhibitory concentrations (MICs) for each isolate were determined following the standard guidelines [12]. Quality control was ensured using *E. coli* ATCC 25922 and *K. pneumoniae* ATCC 700603. MDR was defined as resistance to ≥1 agent in ≥3 antibiotic classes.

### 2.8. Data Management and Analysis

Data were anonymized, double-entered and verified in Microsoft Excel, and then analyzed using GraphPad Prism Version 10.3 and WHONET [13]. To ensure data accuracy, the two independently entered datasets were cross-compared using Excel's cell-by-cell verification function. Any discrepancies identified between entries were reviewed against the original data collection forms and corrected by consensus between two data clerks under the supervision of the investigators. After that, descriptive statistics were performed to summarize demographic, environmental, and clinical variables. Associations between categorical variables were tested using Fisher's exact test (two-tailed), and effect sizes were expressed as odds ratios (ORs) with 95% CIs. For associations involving categorical distributions across multiple groups (for example, age vs. bacterial species), Cramer's *V* statistic was calculated to assess the strength and direction of association between variables. A 0.5 continuity correction (Haldane–Anscombe adjustment) was applied for tables containing zero cells. Related categories (for example, *moderate/poor household cleanliness*) were combined where necessary to maintain valid cell counts. Variables showing perfect prediction were reported descriptively. Statistical significance was defined as *p* ≤ 0.05.

### 2.9. Ethical Considerations

Ethical approval was obtained from the Jomo Kenyatta University of Agriculture and Technology (JKUAT) Ethical Review Board (JKU/ISERC/02316/1704). Research authorization was granted by the National Commission for Science, Technology, and Innovation (NACOSTI) (License No. NACOSTI/P/25/4173390). Written informed consent was obtained from caregivers prior to participation. For children old enough to understand, verbal assent was also sought in addition to parental consent. Participants were informed of their right to withdraw from the study at any point without any penalty to their medical care. Confidentiality was maintained by anonymizing all data using unique identifiers. Electronic data were stored in a password-protected database, and physical questionnaires and laboratory records were kept in locked cabinets. The study adhered to the principles of the Declaration of Helsinki [14].

## 3. Results

### 3.1. Demographic Characteristics of Children Participants

A total of 216 children aged 0–60 months were enrolled and stratified into five age groups (Table 2). The largest proportion belonged to the 48–60-month age group (58, 26.9%), followed by the youngest group (0–11 months) (53, 19.9%), the 36–47-month group (42, 19.5%), the 24–35-month group (41, 19.0%), and the 12–23-month group (32, 14.8%), respectively (Table 2 and File S1). Overall, the sex distribution revealed a higher proportion of female participants (121, 56.0%) compared to males (95, 44.0%) (Table 2 and File S1).

### 3.2. Prevalence of Enteric Bacterial Infections

A total of 204 enteric bacterial isolates were recovered from stool samples of children aged 0–60 months presenting with diarrhea (Table 3 and File S1). The overall prevalence of enteric bacterial infection among children with diarrhea was 94.4% (95% CI: 90.5–96.9), indicating that nearly all participants yielded at least one bacterial pathogen (Table 3).

As shown in Table 3, *E. coli* was the most frequently isolated organism (38.2%, 95% CI: 31.6–45.1), followed by *S. sonnei* (11.8%, 95% CI: 7.7–17.0), *E. cloacae* (9.8%, 95% CI: 6.1–14.6), and *P. vulgaris* (6.9%, 95% CI: 3.9–11.3). Other enteric pathogens detected at lower frequencies included *K. pneumoniae*, *C. freundii*, and *C. braakii* (each 4.9%, 95% CI: 2.4–8.8), while *S. enterica*, *P. shigelloides*, and *A. hydrophila* each accounted for 3.9% (95% CI: 1.7–7.5) of isolates (Table 3). Rarely detected species included *P. rettgeri* (2.9%, 95% CI: 1.1–6.3), *R. planticola* (2.0%, 95% CI: 0.6–5.2), *R. ornithinolytica* (1.0%, 95% CI: 0.1–3.5), and *C. werkmanii* (1.0%, 95% CI: 0.1–3.5), as shown in Table 3.

Notably, no statistically significant association was observed between bacterial species and age group (*p* > 0.05, Table 3). The corresponding Cramer's *V* values (0.02–0.14) also indicated negligible strength of association, suggesting that the distribution of specific bacterial isolates was largely independent of age among children under 5 years (Table 3).

The overall prevalence of enteric bacterial isolates was nearly identical between sexes, with 101 (49.5%) isolates recovered from male children and 103 (50.5%) from females (Table 4 and File S1).

Fisher's exact test showed no statistically significant association between sex and the distribution of any bacterial species (*p* > 0.05 for all). The estimated ORs (males vs. females) for individual species ranged from 0.24 to 2.11, and all 95% CIs crossed 1, indicating negligible sex-related differences in isolation rates (Table 4).

### 3.3. Sociodemographic Characteristics of Parents/Legal Guardians and Households

The sociodemographic characteristics of the parents or legal guardians of the participating children, as well as their households, are summarized in Table 5. Of the 216 respondents, 122 (56.5%) were female and 94 (43.5%) were male (Table 5 and File S1).

The majority of respondents were aged 20–29 years (70, 32.4%) and 30–39 years (67, 31.0%), whereas 34 (15.7%) were below 20 years and 45 (20.8%) were aged 40 years or older (Table 5). Regarding education, more than half (110, 50.9%) of the parents or guardians had attained tertiary-level education, while 60 (27.8%) had completed secondary education, and 46 (21.3%) had primary-level education (Table 5 and File S1).

With respect to household income, 75 (34.7%) earned between Ksh 20,001 and 30,000, and 56 (25.9%) earned above Ksh 30,000 per month. Employment was predominantly informal (76, 35.2%), followed by business/trade (53, 24.5%), formal employment (45, 20.8%), and farming (42, 19.4%) (Table 5 and File S1).

In terms of household composition, the majority of households comprised 4–6 members (126, 58.3%), while 50 (23.1%) had 1–3 members, and 40 (18.5%) had more than six (Table 5). Concerning cooking fuel, gas was the most commonly used (118, 54.6%), followed by charcoal (80, 37.0%) and electricity (10, 4.6%), and no respondents reported using firewood (Table 5 and File S1).

### 3.4. Environmental and Sanitation Characteristics and Association With Enteric Bacterial Infection

Environmental and sanitation characteristics (File S1) were evaluated to determine their association with enteric bacterial infection, and the findings are presented in Table 6.

Soap availability showed a strong and statistically significant association with infection status (Table 6). Among children from households where soap was consistently available, 63 (64.3%) were infected, compared with 110 (93.2%) from households where soap was intermittently or never available (*p* < 0.0001, Table 6). Children living in households with inconsistent or absent soap availability had 7.4-fold higher odds of infection (OR = 7.4; 95% CI: 3.7–15.0) compared with those where soap was always available (Table 6).

Visible indicators of poor sanitation, such as waste accumulation or stagnant water, were reported in 112 (51.9%) of households and were also significantly associated with infection (Table 6). Among these households, 107 (95.5%) of children tested positive for enteric bacteria, compared with 84 (80.8%) from households without visible sanitation problems (*p* = 0.001; Table 6). The presence of visible poor sanitation increased the odds of infection by nearly fivefold (OR = 4.8; 95% CI: 1.7–13.8; Table 6).

Other environmental factors showed no significant associations with pathogen positivity (Table 6). Infection prevalence did not differ significantly by water source (piped = 101/108, 93.5%; borehole = 53/56, 94.6%; purchased = 48/52, 92.3%; *p* = 0.8769), type of sanitation facility (private flush = 75/84, 89.3%; shared flush = 93/96, 96.9%; pit latrine = 36/41, 87.8%; *p* = 0.0552), waste disposal method (city council collection = 68/76, 89.5%; burned = 56/62, 90.3%; open dumping = 62/78, 79.5%; *p* = 0.1276), or presence of a handwashing facility (present = 78/90, 86.7%; not present = 103/126, 81.7%; *p* = 0.3562), as shown in Table 6.

### 3.5. Public Health and Clinical Characteristics

The public health and clinical characteristics of the children and their households are presented in Table 7 and summarized in File S1. Recent travel within 2 weeks was significantly associated with pathogen positivity (*p* = 0.0166, Table 7). Interestingly, children who had not traveled in the 2 weeks prior to sampling were more likely to be infected than those who had traveled, with 177 (92.7%) of nontraveling children testing positive compared to 19 (76.0%) of those who had traveled (Table 7). The odds of infection were 4.1 times higher (OR = 4.1; 95% CI: 1.4–12.0) among children who had not traveled (Table 7).

Duration of diarrhea was also significantly associated with bacterial infection (*p* = 0.0362, Table 7). Infections occurred in 38 (86.4%) of children with diarrhea lasting less than 24 h and 44 (100.0%) of those with prolonged diarrhea (> 48 h), compared with 117 (91.4%) in the 24–48-h group. Prolonged diarrhea (> 48 h) was a perfect predictor of infection in this cohort (Table 7).

Treatment received during illness showed a strong and statistically significant relationship with infection status (*p* < 0.0001, Table 7). Among children who received any treatment (ORS [oral rehydration solution], zinc, or antibiotics), 165 (99.4%) were pathogen-positive compared with 39 (78.0%) among those who received no treatment. Receiving any form of treatment was associated with 17.6-fold higher odds of infection (OR = 17.6; 95% CI: 5.8–53.5) (Table 7).

Awareness of public health information among caregivers was also significantly related to infection status (*p* = 0.001, Table 7). Pathogen positivity was observed in 115 (89.8%) of children whose caregivers were aware of public health messages, compared with 88 (100.0%) of those whose caregivers were not. Awareness of public health information was therefore a perfect predictor of infection in this cohort (Table 7).

Household hygiene showed a strong association with pathogen positivity (*p* < 0.0001, Table 7). Children from moderately clean or poorly maintained households (143/151, 94.7%) were significantly more likely to be infected compared with those from clean and orderly homes (49/65, 75.4%), corresponding to a 4.6-fold higher risk (OR = 4.6; 95% CI: 2.2–9.7) (Table 7).

Safe drinking water access was another significant predictor (*p* = 0.0004, Table 7). All 106 (100.0%) children from households lacking safe water were infected, compared with 98 (89.1%) from households with safe drinking water. The absence of safe water was associated with 12.9-fold higher odds of infection (OR = 12.9; 95% CI: 1.7–98.9).

No significant associations were found between enteric bacterial infection and feeding practice (*p* = 0.5565), vaccination status (*p* = 0.9168), frequency of diarrheal episodes (*p* = 0.3671), access to health services (*p* = 0.1054), or satisfaction with health services (*p* = 0.2763) (Table 7).

Overall, these findings suggest that treatment type, household hygiene, and water safety were the strongest predictors of enteric bacterial infection, whereas feeding practices, vaccination, and healthcare access did not show significant effects (Table 7).

### 3.6. Antimicrobial Susceptibility Profiles of Enteric Bacterial Isolates

The antimicrobial susceptibility profiles of the 204 enteric bacterial isolates are presented in Table 8. In this study, meropenem demonstrated complete activity, with all 204 (100.0%) isolates susceptible (Table 8 and File S1). Similarly, amikacin showed full susceptibility across all 204 (100.0%) isolates (Table 8). Gentamicin retained high activity, with 75/78 (96.2%) *E. coli*, all (20, 100.0%) *E. cloacae*, and all other species showing complete susceptibility (Table 8). Ciprofloxacin exhibited a comparable susceptibility pattern, with 75/78 (96.2%) *E. coli* isolates and all (100.0%) of the remaining species susceptible (Table 8).

Ceftriaxone maintained moderately high activity, with 49/78 (62.8%) *E. coli*, all (8, 100.0%) *S. enterica*, and *E. cloacae* (20, 100.0%) isolates demonstrating susceptibility (Table 8).

In contrast, ampicillin displayed reduced activity across several bacterial groups as shown in Table 8. Resistance to ampicillin was observed in 63/78 (80.8%) *E. coli*, all (24, 100.0%) *S. sonnei*, and 10/14 (71.4%) *P. vulgaris* isolates (Table 8). Similarly, amoxicillin–clavulanic acid showed poor activity, with 18/20 (90.0%) *E. cloacae* and 9/10 (90.0%) *C. freundii* isolates demonstrating resistance (Table 8).

Nitrofurantoin exhibited variable susceptibility across species (Table 8). *E. coli* showed 54/78 (69.2%) susceptible isolates, *S. sonnei* 6/24 (25.0%), and *P. vulgaris* 6/14 (42.9%), whereas *K. pneumoniae* and *E. cloacae* were fully susceptible (Table 8).

### 3.7. AMR and MDR Patterns of the Bacterial Isolates

The AMR and MDR patterns of the 204 enteric bacterial isolates are summarized in Table 9 and File S1. Overall, 169/204 (82.8%) isolates exhibited resistance to at least one antimicrobial agent, while 119/204 (58.3%) met the criteria for MDR (Table 9).

Among *E. coli* isolates, 69/78 (88.5%) were resistant to at least one antimicrobial, and 52/78 (66.7%) were MDR (Table 9). Similarly, *S. sonnei* showed 23/24 (95.8%) AMR and 16/24 (66.7%) MDR. *C. braakii* demonstrated 10/10 (100.0%) AMR and 8/10 (80.0%) MDR, while *A. hydrophila* exhibited 8/8 (100.0%) AMR and 6/8 (75.0%) MDR (Table 9).

High MDR levels were also recorded in *C. freundii* (7/10, 70.0%), *P. vulgaris* (8/14, 57.1%), *E. cloacae* (10/20, 50.0%), *P. rettgeri* (3/6, 50.0%), and *P. shigelloides* (5/8, 62.5%) (Table 9). In contrast, no MDR was detected in *S. enterica* (0/8, 0.0%), *R. planticola* (0/4, 0.0%), *R. ornithinolytica* (0/2, 0.0%), or *C. werkmanii* (0/2, 0.0%) (Table 9).

Overall, MDR was observed across multiple bacterial genera, with the highest proportions recorded in *C. braakii*, *A. hydrophila*, and *C. freundii* (Table 9).

## 4. Discussion

### 4.1. Epidemiological Significance

This study provides a comprehensive understanding of the bacterial etiology of childhood diarrhea in Nairobi County and reveals patterns comparable to those reported across sub-Saharan Africa. Similar to findings from Tanzania [15] and Ethiopia [16], where bacterial pathogens accounted for between 40% and 80% of diarrheal cases, this study reinforces the continued predominance of bacterial infections among children under 5 years. However, the higher prevalence observed in the present cohort may reflect enhanced pathogen recovery due to enrichment culturing and automated identification, which increase sensitivity compared to conventional methods.

The dominance of *Escherichia coli* mirrors reports from Kenya [17], Uganda [18], and Nigeria [19], where *E. coli* remains a leading pediatric enteric pathogen. However, the emergence of *Shigella sonnei* as a major *Shigella* species in this study contrasts with older East African data, which predominantly identified *S. flexneri* [20]. This shift is consistent with observations in South Africa [21] and parts of Asia [22], where *S. sonnei* has replaced *S. flexneri*, likely due to improved water chlorination and changing sanitation conditions that favor more chlorine-resistant species. The detection of opportunistic bacteria such as *Enterobacter cloacae*, *Proteus vulgaris*, and *Aeromonas hydrophila* also aligns with studies from Ghana [23] and India [24], suggesting that environmental contamination and poor waste management contribute significantly to pathogen exposure in children.

This evolving epidemiological landscape underscores the need for continuous local surveillance of enteric pathogens. Urbanization, rapid population growth, and unregulated waste disposal sustain reservoirs of enteric bacteria, facilitating recurrent outbreaks. Moreover, the identification of emerging and environmental species implies that traditional diagnostic approaches may underestimate the diversity of diarrheal pathogens. Incorporating molecular diagnostic tools and genomic sequencing in future studies could improve detection and inform more targeted interventions against both classical and emerging enteric agents.

### 4.2. Environmental and Socioeconomic Determinants

The strong relationship between infection risk, environmental conditions, and socioeconomic context aligns with similar studies conducted in Malawi [25], Bangladesh [26], and Ethiopia [27], all of which highlight the central role of poverty, overcrowding, and inadequate sanitation in sustaining transmission. The higher infection burden among older children is consistent with findings from Ghana [28] and Nigeria [29], where increased environmental exposure and mobility were linked to elevated risk. The absence of significant sex differences in infection prevalence is also in agreement with previous reports [30], affirming that behavioral rather than biological factors drive infection dynamics.

The coexistence of relatively high caregiver education levels with persistent diarrheal burden has been similarly reported in studies from Rwanda [31] and Tanzania [32], suggesting that knowledge alone is insufficient to reduce infection risk when households face structural barriers such as economic hardship, intermittent water supply, or limited access to sanitation. These findings emphasize that behavioral change interventions must be supported by enabling environments that allow caregivers to practice safe hygiene consistently.

These observations reinforce conclusions drawn in global WASH research [28, 29, 31–33], indicating that infrastructure alone does not guarantee improved health outcomes. The quality, reliability, and consistent use of sanitation and hygiene resources remain pivotal. For instance, a Ugandan study [34] found that households with functional handwashing stations but without soap had similar diarrhea rates as those with no handwashing facility at all. These findings collectively demonstrate that diarrheal control strategies must integrate infrastructural improvements with social, economic, and behavioral interventions to achieve meaningful disease reduction.

### 4.3. Clinical and Treatment Implications

The clinical presentation patterns observed in this study align closely with findings from other East African research. Studies in Tanzania [35] and Uganda [36] have reported similar symptom profiles—dominated by abdominal pain, fever, and bloody stool—indicating the predominance of invasive bacterial pathogens such as *Shigella* and diarrheagenic *E. coli*. Comparable observations were made in Nigeria [37], where disease severity correlated with both delayed healthcare seeking and limited access to appropriate antimicrobial therapy.

Suboptimal adherence to WHO-recommended management practices has been observed widely across Africa. Low use of ORS and zinc supplements mirrors patterns in Ethiopia [38] and Ghana [39], where caregivers often resort to antibiotics for diarrhea management. These trends stem from limited caregiver education on treatment protocols, supply shortages in local clinics, and the perception that antibiotics provide faster relief. The inappropriate use of antibiotics not only contributes to the escalation of AMR but also delays effective rehydration therapy, which remains the cornerstone of diarrhea management.

These findings collectively highlight systemic gaps in both community and clinical diarrhea management. Studies in Kenya [40] and Malawi [41] have shown that interventions led by community health workers can significantly improve ORS and zinc uptake while reducing unnecessary antibiotic use. Strengthening such community-based initiatives, alongside continuous provider training and reliable supply chains, could bridge the gap between knowledge and practice and enhance adherence to evidence-based management of childhood diarrhea.

### 4.4. AMR and Stewardship

The AMR profile observed in this is consistent with regional and global evidence of increasing resistance among enteric pathogens. Similar resistance to first-line antibiotics such as ampicillin and amoxicillin–clavulanic acid has been reported in Tanzania [39], Uganda [42], and India [43], underscoring the widespread erosion of efficacy of commonly prescribed agents. The high prevalence of MDR mirrors results from Ethiopia [44] and Ghana [45], where over half of *E. coli* and *Shigella* isolates exhibited resistance to three or more antibiotic classes.

Comparatively, some East African studies have reported slightly lower MDR rates [46], possibly reflecting regional differences in antibiotic availability, prescription patterns, and infection control practices. The detection of resistant environmental species such as *Aeromonas hydrophila* and *Enterobacter cloacae* parallels findings from Nigeria [47] and Bangladesh [48], highlighting the environmental spread of resistance genes through wastewater and food contamination.

The retained susceptibility to meropenem, ciprofloxacin, and amikacin is in agreement with reports from Rwanda [49] and Uganda [50], confirming their continuing utility as last-line therapies. However, similar to other reports [15, 51], the near-complete inefficacy of ampicillin and *β*-lactam/*β*-lactamase inhibitor combinations underscores the urgent need for stewardship. Integrating AMR surveillance within pediatric diarrhea programs, strengthening prescription regulation, and improving laboratory diagnostic capacity are critical steps toward containing resistance spread in the region.

### 4.5. Public Health and Policy Implications

The convergence of high bacterial prevalence, socioeconomic inequities, and extensive AMR presents a multifaceted public health crisis that threatens to undermine progress toward Sustainable Development Goal 3.2 on child health. The patterns observed here echo those reported in other urban African settings, including Accra [52], Dar es Salaam [53], and Kampala [54], where diarrheal disease remains entrenched despite improvements in sanitation coverage.

At the clinical level, expanding diagnostic capacity to include routine stool culture and antimicrobial susceptibility testing could substantially reduce empirical antibiotic use and improve treatment outcomes. In Malawi and South Africa [55, 56], integrating culture-based diagnostics into outpatient pediatric care improved antimicrobial targeting and reduced overall antibiotic prescription rates.

At the community level, behavior-centered WASH interventions have shown remarkable success. In Bangladesh [57] and Kenya [58], interventions combining hygiene education with subsidized soap and water treatment supplies achieved sustained reductions in diarrhea incidence. The present findings therefore support the integration of WASH promotion into maternal and child health programs to maximize reach and sustainability.

At the policy level, this study reinforces the “One Health” perspective, which recognizes the interconnectedness of human, animal, and environmental health in tackling AMR and enteric infections. Experiences from Rwanda [59] and Kenya [40] demonstrate that national AMR action plans can only succeed when environmental and food safety sectors are included alongside clinical and pharmaceutical regulation. Strengthening intersectoral collaboration, community surveillance, and cross-border information sharing is vital for sustained AMR control.

Ultimately, reducing diarrheal morbidity and resistance prevalence requires systemic investments in WASH infrastructure, antibiotic regulation, and health system strengthening. As other studies in Kenya [60] and Tanzania [61] have shown, these measures not only prevent infections but also reduce the selective pressures that drive resistance. A coordinated One Health framework that unites clinicians, policymakers, and communities is essential to preserve antimicrobial efficacy and safeguard child health for future generations.

## 5. Conclusion

This study demonstrates a high prevalence of enteric bacterial infections (94.4%) and alarming levels of AMR (82.8%) and MDR (58.3%) among under-five children in Nairobi County. The findings highlight the critical influence of environmental hygiene, sanitation, and water safety on infection risk, emphasizing that consistent access to safe drinking water and soap is a key preventive measure. The poor performance of commonly used antibiotics underscores the urgent need for antimicrobial stewardship and strengthened laboratory-based surveillance to guide empirical therapy. Targeted public health interventions focusing on improved WASH infrastructure, rational antibiotic use, and community education are imperative to reduce disease burden and curb the spread of resistant pathogens.

## Figures and Tables

**Table 1:** Sampling method.

**Facility**	**Estimated monthly caseload**	**Sample (** **N** **)**
Gertrude's Children's Hospital	300	106
Mbagathi District Hospital	100	35
Kahawa West Health Centre	50	18
Westlands Health Centre	50	18
Korogocho Health Centre	50	18
Mathare North Health Centre	60	21

**Table 2:** Demographic characteristics of children aged 0–60 months enrolled in this study.

**Age group (months)**	**Sex**	**Frequency (** **n** **)**	**Percentage (%)**
0–11 months	Female	16	7.4%
	Male	27	12.5%
12–23 months	Female	20	9.3%
	Male	12	5.6%
24–35 months	Female	29	13.4%
	Male	12	5.6%
36–47 months	Female	30	13.9%
	Male	12	5.6%
48–60 months	Female	26	12.0%
	Male	32	14.8%
Total		216	100.0%

**Table 3:** Prevalence of enteric bacterial isolates by age group among children aged 5 years and below.

**Isolate (** **n** **)**	**Status**	**Age group (months)**					**Cramer's ** **V**	**% prevalence (95% CI)**	**p** ** value**
		**0–11 (** **n** **, %)**	**12–23 (** **n** **, %)**	**24–35 (** **n** **, %)**	**36–47 (** **n** **, %)**	**48–60 (** **n** ** , %)**			
*E. coli* (*n* = 78)	*Positive*	7 (9.0%)	12 (15.4%)	20 (25.6%)	15 (19.2%)	20 (25.6%)	0.11	38.2 (31.6–45.1)	0.1283
	*Negative*	1 (1.3%)	2 (2.6%)	0 (0.0%)	1 (1.3%)	0 (0.0%)			
*S. sonnei* (*n* = 24)	*Positive*	0 (0.0%)	3 (12.5%)	5 (20.8%)	5 (20.8%)	8 (33.3%)	0.09	11.8 (7.7–17.0)	0.5020
	*Negative*	0 (0.0%)	1 (4.2%)	1 (4.2%)	1 (4.2%)	0 (0.0%)			
*K. pneumoniae* (*n* = 10)	*Positive*	2 (20.0%)	2 (20.0%)	1 (10.0%)	2 (20.0%)	2 (20.0%)	0.06	4.9 (2.4–8.8)	> 0.9999
	*Negative*	0 (0.0%)	0 (0.0%)	1 (10.0%)	0 (0.0%)	0 (0.0%)			
*S. enterica* (*n* = 8)	*Positive*	1 (12.5%)	1 (12.5%)	1 (12.5%)	2 (25.0%)	2 (25.0%)	0.05	3.9 (1.7–7.5)	> 0.9999
	*Negative*	0 (0.0%)	0 (0.0%)	1 (12.5%)	0 (0.0%)	0 (0.0%)			
*P. vulgaris* (*n* = 14)	*Positive*	3 (21.4%)	1 (7.1%)	2 (14.3%)	3 (21.4%)	3 (21.4%)	0.07	6.9 (3.9–11.3)	> 0.9999
	*Negative*	0 (0.0%)	0 (0.0%)	1 (7.1%)	0 (0.0%)	1 (7.1%)			
*E. cloacae* (*n* = 20)	*Positive*	2 (10.0%)	4 (20.0%)	4 (20.0%)	3 (15.0%)	5 (25.0%)	0.10	9.8 (6.1–14.6)	> 0.9999
	*Negative*	0 (0.0%)	0 (0.0%)	0 (0.0%)	1 (5.0%)	1 (5.0%)			
*R. planticola* (*n* = 4)	*Positive*	1 (25.0%)	0 (0.0%)	0 (0.0%)	1 (25.0%)	1 (25.0%)	0.08	2.0 (0.6–5.2)	> 0.9999
	*Negative*	0 (0.0%)	0 (0.0%)	1 (25.0%)	0 (0.0%)	0 (0.0%)			
*R. ornithinolytica* (*n* = 2)	*Positive*	0 (0.0%)	0 (0.0%)	0 (0.0%)	1 (50.0%)	0 (0.0%)	0.10	1.0 (0.1–3.5)	> 0.9999
	*Negative*	0 (0.0%)	0 (0.0%)	1 (50.0%)	0 (0.0%)	0 (0.0%)			
*P. rettgeri* (*n* = 6)	*Positive*	0 (0.0%)	0 (0.0%)	0 (0.0%)	2 (33.3%)	3 (50.0%)	0.12	2.9 (1.1–6.3)	> 0.9999
	*Negative*	0 (0.0%)	0 (0.0%)	0 (0.0%)	1 (16.7%)	0 (0.0%)			
*P. shigelloides* (*n* = 8)	*Positive*	2 (25.0%)	0 (0.0%)	0 (0.0%)	3 (37.5%)	2 (25.0%)	0.09	3.9 (1.7–7.5)	> 0.9999
	*Negative*	0 (0.0%)	0 (0.0%)	0 (0.0%)	1 (12.5%)	0 (0.0%)			
*C. werkmanii* (*n* = 2)	*Positive*	0 (0.0%)	1 (50.0%)	0 (0.0%)	0 (0.0%)	1 (50.0%)	0.14	1.0 (0.1–3.5)	ND
	*Negative*	0 (0.0%)	0 (0.0%)	0 (0.0%)	0 (0.0%)	0 (0.0%)			
*C. freundii* (*n* = 10)	*Positive*	4 (40.0%)	0 (0.0%)	1 (10.0%)	1 (10.0%)	2 (20.0%)	0.13	4.9 (2.4–8.8)	0.3333
	*Negative*	0 (0.0%)	0 (0.0%)	1 (10.0%)	1 (10.0%)	0 (0.0%)			
*C. braakii* (*n* = 10)	*Positive*	0 (0.0%)	0 (0.0%)	2 (20.0%)	3 (30.0%)	4 (40.0%)	0.12	4.9 (2.4–8.8)	0.6000
	*Negative*	0 (0.0%)	0 (0.0%)	1 (10.0%)	0 (0.0%)	0 (0.0%)			
*A. hydrophila* (*n* = 8)	*Positive*	0 (0.0%)	2 (25.0%)	1 (12.5%)	2 (25.0%)	2 (25.0%)	0.08	3.9 (1.7–7.5)	> 0.9999
	*Negative*	0 (0.0%)	0 (0.0%)	0 (0.0%)	0 (0.0%)	1 (12.5%)			
Total (*n* = 204)		23 (11.3%)	29 (14.2%)	45 (22.1%)	49 (24.0%)	58 (28.4%)		94.4 (90.5–96.9)	

*Note:* Statistical analysis was performed using Fisher's exact test, and significance was considered at *p* < 0.05. ND: not determined due to small sample size. 95% CIs: exact binomial (Clopper–Pearson) intervals.

**Table 4:** Prevalence of enteric bacterial isolates by sex among children aged 5 years and below.

**Isolate**	**Total isolates**	**Male ** **n** ** (%)**	**Female ** **n** ** (%)**	**OR (95% CI)**	**p** ** value**
*E. coli*	78	35 (44.9)	40 (51.3)	0.79 (0.43–1.45)	> 0.9999
*S. sonnei*	24	14 (58.3)	9 (37.5)	1.73 (0.67–4.49)	0.4167
*K. pneumoniae*	10	6 (60.0)	3 (30.0)	2.11 (0.47–9.43)	> 0.9999
*S. enterica*	8	4 (50.0)	3 (37.5)	1.37 (0.29–6.46)	> 0.9999
*P. vulgaris*	14	4 (28.6)	7 (50.0)	0.55 (0.15–2.03)	0.5385
*E. cloacae*	20	9 (45.0)	8 (40.0)	1.17 (0.43–3.18)	0.2421
*R. planticola*	4	1 (25.0)	2 (50.0)	0.50 (0.04–5.96)	> 0.9999
*R. ornithinolytica*	2	1 (50.0)	1 (50.0)	1.02 (0.06–16.77)	> 0.9999
*P. rettgeri*	6	1 (16.7)	4 (66.7)	0.24 (0.03–2.10)	0.3333
*P. shigelloides*	8	3 (37.5)	4 (50.0)	0.75 (0.16–3.41)	> 0.9999
*C. werkmanii*	2	1 (50.0)	1 (50.0)	1.02 (0.06–16.77)	> 0.9999
*C. freundii*	10	3 (30.0)	6 (60.0)	0.48 (0.12–1.96)	0.4000
*C. braakii*	10	2 (20.0)	6 (60.0)	0.32 (0.06–1.69)	> 0.9999
*A. hydrophila*	8	4 (50.0)	3 (37.5)	1.38 (0.29–6.62)	> 0.9999
Total	204	101 (49.5)	103 (50.5)	—	—

*Note:* Statistical analysis was performed using Fisher's exact test, and significance was considered at *p* < 0.05.

**Table 5:** Sociodemographic characteristics of parents/legal guardians.

**Variable**	**Category**	**Frequency (** **n** **)**	**Percentage (%)**
Sex of parent	Male	94	43.5
	Female	122	56.5

Age of parent (years)	< 20	34	15.7
	20–29	70	32.4
	30–39	67	31.0
	≥ 40	45	20.8

Level of education	No informal education	0	0.0
	Primary education	46	21.3
	Secondary education	60	27.8
	Tertiary education	110	50.9

Monthly household income (KES)	< 10,000	25	11.6
	10,000–20,000	60	27.8
	20,001–30,000	75	34.7
	> 30,001	56	25.9

Type of employment	Formal employment	45	20.8
	Informal employment	76	35.2
	Business/trade	53	24.5
	Farming	42	19.4

Household size	1–3 members	50	23.1
	4–6 members	126	58.3
	> 6 members	40	18.5

Cooking fuel	Electricity	10	4.6
	Gas	118	54.6
	Charcoal	80	37.0
	Firewood	0	0.0

*Note: n* = 216.

**Table 6:** Environmental and sanitation characteristics and association with diarrheagenic enteric bacterial pathogen infection.

**Variable**	**Category**	**Pathogen positive, ** **n** ** (%)**	**Pathogen negative, ** **n** ** (%)**	**p** ** value**	**OR [95% CI]**
Water source	Piped water	101 (93.5%)	7 (6.5%)	0.8769	Ref
	Borehole	53 (94.6%)	3 (5.4%)		1.3 [0.3–5.1]
	Purchased water	48 (92.3%)	4 (7.7%)		0.8 [0.2–3.0]

Sanitation facility	Private flush toilet	75 (89.3%)	9 (10.7%)	0.0552	Ref
	Shared flush toilet	93 (96.9%)	3 (3.1%)		3.7 [1.0–14.1]
	Pit latrine	36 (87.8%)	5 (12.2%)		0.9 [0.3–2.8]

Waste disposal method	City council collection	68 (89.5%)	8 (10.5%)	0.1276	Ref
	Burned	56 (90.3%)	6 (9.7%)		1.1 [0.4–3.3]
	Dumped in open	62 (79.5%)	16 (20.5%)		0.5 [0.2–1.2]

Handwashing facility	Present	78 (86.7%)	12 (13.3%)	0.3562	Ref
	Not present	103 (81.7%)	23 (18.3%)		0.7 [0.3–1.5]

Soap availability	Always	63 (64.3%)	35 (35.7%)	< 0.0001	Ref
	Sometimes/never	110 (93.2%)	8 (6.8%)		7.4 [3.7–15.0]

Visible poor sanitation	Yes	107 (95.5%)	5 (4.5%)	0.001	4.8 [1.7–13.8]
	No	84 (80.8%)	20 (19.2%)		Ref

*Note:* Data are presented as frequencies and row percentages. Statistical analysis was performed using Fisher's exact test, and significance was considered at *p* < 0.05.

Abbreviations: CI, confidence interval; OR, odds ratio; Ref, reference category.

**Table 7:** Public health and clinical characteristics and their association with diarrheagenic enteric bacterial infection.

**Variable**	**Category**	**Pathogen positive ** **n** ** (%)**	**Pathogen negative ** **n** ** (%)**	**p** ** value**	**OR [95% CI]**
Feeding practice	Exclusively breastfed	74 (89.2%)	11 (13.3%)	0.5565	Ref
	Mixed/formula	108 (82.4%)	23 (17.6%)		0.6 [0.3–1.3]

Vaccination status	Fully vaccinated	155 (85.2%)	27 (14.8%)	0.9168	Ref
	Partial/not	29 (85.3%)	5 (14.7%)		1.0 [0.4–2.8]

Recent travel	Yes	19 (76.0%)	6 (24.0%)	0.0166	Ref
	No	177 (92.7%)	14 (7.3%)		4.1 [1.4–12.0]

Diarrhea duration	24–48 h	117 (91.4%)	11 (8.6%)	0.0362	Ref
	< 24 h	38 (86.4%)	6 (13.6%)		0.6 [0.2–1.8]
	> 48 h	44 (100.0%)	0 (0.0%)		∞ [Undefined]

Treatment received	None	39 (78.0%)	11 (22.0%)	< 0.0001	Ref
	Any treatment^a^	165 (99.4%)	1 (0.6%)		17.6 [5.8–53.5]

Diarrheal episodes (12 months)	1–2 times	32 (94.1%)	2 (5.9%)	0.3671	Ref
	3–5 times	106 (98.1%)	2 (1.9%)		2.7 [0.4–19.6]
	> 5 times	73 (98.6%)	1 (1.4%)		3.6 [0.3–41.4]

Awareness of public health	Yes	115 (89.8%)	13 (10.2%)	0.001	Ref
	No	88 (100.0%)	0 (0.0%)		∞ [Undefined]

Access to health services	Easy/moderate	118 (92.2%)	10 (7.8%)	0.1054	Ref
	Difficult/no access	87 (98.9%)	1 (1.1%)		5.9 [0.7–47.2]

Satisfaction with services	Satisfied^b^	127 (84.1%)	24 (15.9%)	0.2763	Ref
	Not satisfied	60 (92.3%)	5 (7.7%)		2.3 [0.8–6.3]

Household hygiene	Clean and orderly	49 (75.4%)	16 (24.6%)	< 0.0001	Ref
	Moderate/poor	143 (94.7%)	8 (5.3%)		4.6 [2.2–9.7]

Safe drinking water	Yes	98 (89.1%)	12 (10.9%)	0.0004	Ref
	No	106 (100.0%)	0 (0.0%)		12.9 [1.7–98.9]

*Note:* Data are presented as frequencies and row percentages. Statistical analysis was performed using Fisher's exact test and significance was considered at *p* < 0.05.

Abbreviations: CI, confidence interval; OR, odds ratio; Ref, reference category.

^a^Any treatment: ORS, zinc, or antibiotics.

^b^Satisfied: very satisfied and somewhat satisfied combined.

**Table 8:** Antimicrobial susceptibility profile of the recovered bacterial isolates.

**Isolate**	**CTX**		**AMP**		**GEN**		**MRP**		**AMK**		**CPX**		**NTF**		**AMC**	
	**S**	**R**	**S**	**R**	**S**	**R**	**S**	**R**	**S**	**R**	**S**	**R**	**S**	**R**	**S**	**R**
*E. coli (n* = 78)	49 (62.8%)	29 (37.2%)	15 (19.2%)	63 (80.8%)	75 (96.2%)	3 (3.8%)	78 (100.0%)	0 (0.0%)	5 (6.4%)	73 (93.6%)	3 (3.8%)	75 (96.2%)	0 (0.0%)	78 (100.0%)	54 (69.2%)	24 (30.8%)
*S. sonnei (n* = 24)	18 (75.0%)	6 (25.0%)	0 (0.0%)	24 (100.0%)	24 (100.0%)	0 (0.0%)	24 (100.0%)	0 (0.0%)	0 (0.0%)	24 (100.0%)	0 (0.0%)	24 (100.0%)	6 (25.0%)	18 (75.0%)	18 (75.0%)	6 (25.0%)
*K. pneumoniae (n* = 10)	10 (100.0%)	0 (0.0%)	4 (40.0%)	6 (60.0%)	10 (100.0%)	0 (0.0%)	10 (100.0%)	0 (0.0%)	10 (100.0%)	0 (0.0%)	10 (100.0%)	0 (0.0%)	10 (100.0%)	0 (0.0%)	2 (20.0%)	8 (80.0%)
*S. enterica (n* = 8)	8 (100.0%)	0 (0.0%)	0 (0.0%)	8 (100.0%)	8 (100.0%)	0 (0.0%)	8 (100.0%)	0 (0.0%)	8 (100.0%)	0 (0.0%)	8 (100.0%)	0 (0.0%)	0 (0.0%)	8 (100.0%)	6 (75.0%)	2 (25.0%)
*P. vulgaris (n* = 14)	14 (100.0%)	0 (0.0%)	4 (28.6%)	10 (71.4%)	14 (100.0%)	0 (0.0%)	14 (100.0%)	0 (0.0%)	14 (100.0%)	0 (0.0%)	14 (100.0%)	0 (0.0%)	14 (100.0%)	0 (0.0%)	6 (42.9%)	8 (57.1%)
*E. cloacae (n* = 20)	20 (100.0%)	0 (0.0%)	7 (35.0%)	13 (65.0%)	20 (100.0%)	0 (0.0%)	20 (100.0%)	0 (0.0%)	20 (100.0%)	0 (0.0%)	20 (100.0%)	0 (0.0%)	20 (100.0%)	0 (0.0%)	2 (10.0%)	18 (90.0%)
*R. planticola (n* = 4)	4 (100.0%)	0 (0.0%)	0 (0.0%)	4 (100.0%)	4 (100.0%)	0 (0.0%)	4 (100.0%)	0 (0.0%)	4 (100.0%)	0 (0.0%)	4 (100.0%)	0 (0.0%)	4 (100.0%)	0 (0.0%)	4 (100.0%)	0 (0.0%)
*R. ornithinolytica (n* = 2)	2 (100.0%)	0 (0.0%)	0 (0.0%)	2 (100.0%)	2 (100.0%)	0 (0.0%)	2 (100.0%)	0 (0.0%)	2 (100.0%)	0 (0.0%)	2 (100.0%)	0 (0.0%)	2 (100.0%)	0 (0.0%)	0 (0.0%)	2 (100.0%)
*P. rettgeri (n* = 6)	6 (100.0%)	0 (0.0%)	3 (50.0%)	3 (50.0%)	6 (100.0%)	0 (0.0%)	6 (100.0%)	0 (0.0%)	6 (100.0%)	0 (0.0%)	6 (100.0%)	0 (0.0%)	6 (100.0%)	0 (0.0%)	3 (50.0%)	3 (50.0%)
*P. shigelloides (n* = 8)	8 (100.0%)	0 (0.0%)	3 (37.5%)	5 (62.5%)	8 (100.0%)	0 (0.0%)	8 (100.0%)	0 (0.0%)	8 (100.0%)	0 (0.0%)	8 (100.0%)	0 (0.0%)	8 (100.0%)	0 (0.0%)	2 (25.0%)	6 (75.0%)
*C. werkmanii (n* = 2)	2 (100.0%)	0 (0.0%)	1 (50.0%)	1 (50.0%)	2 (100.0%)	0 (0.0%)	2 (100.0%)	0 (0.0%)	2 (100.0%)	0 (0.0%)	2 (100.0%)	0 (0.0%)	2 (100.0%)	0 (0.0%)	1 (50.0%)	1 (50.0%)
*C. freundii (n* = 10)	10 (100.0%)	0 (0.0%)	6 (60.0%)	4 (40.0%)	10 (100.0%)	0 (0.0%)	10 (100.0%)	0 (0.0%)	10 (100.0%)	0 (0.0%)	10 (100.0%)	0 (0.0%)	10 (100.0%)	0 (0.0%)	1 (10.0%)	9 (90.0%)
*C. braakii (n* = 10)	10 (100.0%)	0 (0.0%)	8 (80.0%)	2 (20.0%)	10 (100.0%)	0 (0.0%)	10 (100.0%)	0 (0.0%)	10 (100.0%)	0 (0.0%)	10 (100.0%)	0 (0.0%)	10 (100.0%)	0 (0.0%)	0 (0.0%)	10 (100.0%)
*A. hydrophila (n* = 8)	8 (100.0%)	0 (0.0%)	0 (0.0%)	8 (100.0%)	8 (100.0%)	0 (0.0%)	8 (100.0%)	0 (0.0%)	8 (100.0%)	0 (0.0%)	8 (100.0%)	0 (0.0%)	8 (100.0%)	0 (0.0%)	0 (0.0%)	8 (100.0%)
**Standard isolates**																
*E. coli* (ATCC 25922)	✓	✗	✓	✗	✓	✗	✓	✗	✓	✗	✓	✗	✓	✗	✓	✗
*S. typhimurium* (ATCC 14028)	✓	✗	✗	✓	✓	✗	✓	✗	✓	✗	✓	✗	✓	✗	✗	✓
*C. freundii* (ATCC 8090)	✓	✗	✗	✓	✓	✗	✓	✗	✓	✗	✓	✗	✓	✗	✓	✗
*S. flexneri ATCC 12022*	✓	✗	✓	✗	✓	✗	✓	✗	✓	✗	✓	✗	✓	✗	✓	✗
*P. vulgaris (ATCC 6380)*	✓	✗	✗	✓	✓	✗	✓	✗	✓	✗	✓	✗	✓	✗	✓	✗

*Note:* ✓: yes; ✗: no.

Abbreviations: AMC, amoxicillin–clavulanic acid; AMK, amikacin; AMP, ampicillin; CPX, ciprofloxacin; CTX, ceftriaxone; GEN, gentamycin; MRP, meropenem; NTF, nitrofurantoin; R, resistant; S, susceptible.

**Table 9:** Prevalence of AMR and MDR among bacterial isolates from the study participants.

**Bacterial species**	**Total isolates (** **n** **)**	**No. AMR isolates (%)**	**No. MDR isolates (%)**
*E. coli*	78	69 (88.5%)	52 (66.7%)
*S. sonnei*	24	23 (95.8%)	16 (66.7%)
*K. pneumoniae*	10	6 (60.0%)	4 (40.0%)
*S. enterica*	8	2 (25.0%)	0 (0.0%)
*P. vulgaris*	14	10 (71.4%)	8 (57.1%)
*E. cloacae*	20	13 (65.0%)	10 (50.0%)
*R. planticola*	4	4 (100.0%)	0 (0.0%)
*R. ornithinolytica*	2	2 (100.0%)	0 (0.0%)
*P. rettgeri*	6	6 (100.0%)	3 (50.0%)
*P. shigelloides*	8	6 (75.0%)	5 (62.5%)
*C. werkmanii*	2	1 (50.0%)	0 (0.0%)
*C. freundii*	10	9 (90.0%)	7 (70.0%)
*C. braakii*	10	10 (100.0%)	8 (80.0%)
*A. hydrophila*	8	8 (100.0%)	6 (75.0%)
Total	204	169 (82.8%)	119 (58.3%)

*Note:* AMR (antimicrobial resistance): presence of resistance to at least one antimicrobial agent. MDR (multidrug resistance): resistance to at least one agent in ≥ 3 different antimicrobial classes.

## Data Availability

Due to ethical and confidentiality considerations, the full dataset supporting this study is not publicly available; however, a deidentified summary dataset and the isolate resistance profiles are provided as supporting information (File S1).

## Nomenclature

AMK: amikacin
AMP: ampicillin
AMR: antimicrobial resistance
AST: antimicrobial susceptibility testing
ATCC: American Type Culture Collection
CFU: colony-forming unit
CLSI: Clinical and Laboratory Standards Institute
CTX: ceftriaxone
DNA: deoxyribonucleic acid
EPEC: enteropathogenic *Escherichia coli*
ETEC: enterotoxigenic *Escherichia coli*
GEN: gentamicin
IMViC: indole, methyl red, Voges-Proskauer, citrate
KUTRH: Kenyatta University Teaching, Research, and Referral Hospital
LMICs: low- and middle-income countries
MDR: multidrug resistance
MIC: minimum inhibitory concentration
MRP: meropenem
NACOSTI: National Commission for Science, Technology, and Innovation
NIT: nitrofurantoin
ORS: oral rehydration solution
PBS: phosphate-buffered saline
*SS*: *Salmonella–Shigella*
TSI: triple sugar iron
UNICEF: United Nations Children's Fund
VITEK2: automated microbial identification and antimicrobial susceptibility testing system (bioMérieux)
WASH: water, sanitation, and hygiene
WHO: World Health Organization
WHONET: World Health Organization's Laboratory Data Management Software

## Supporting Information

Supporting Information Additional supporting information can be found online in the Supporting Information section. File S1. Supporting information containing summary demographic, risk factor, clinical, and antimicrobial susceptibility data.
